# Gene variation in IL-7 receptor (IL-7R)α affects IL-7R response in CD4+ T cells in HIV-infected individuals

**DOI:** 10.1038/srep42036

**Published:** 2017-02-09

**Authors:** Hans Jakob Hartling, Lars P. Ryder, Henrik Ullum, Niels Ødum, Susanne Dam Nielsen

**Affiliations:** 1 Viro-Immunology Research Unit, Department of Infectious Diseases, Rigshospitalet, Copenhagen University Hospital, Denmark; 2 Department of Clinical Immunology, Rigshospitalet, Copenhagen University Hospital, Denmark; 3 Department of Immunology and Microbiology, Faculty of Health and Medical Sciences, University of Copenhagen, Copenhagen, Denmark

## Abstract

Optimal CD4+ T cell recovery after initiating combination antiretroviral treatment (cART) in HIV infection reduces risk of morbidity and mortality. T-allele homozygosity (‘TT’) in the single nucleotide polymorphism, rs6897932(C/T), in the IL-7 receptor α (IL-7RA) is associated with faster CD4+ T cell recovery after cART initiation compared to C-allele homozygosity in rs6897932 (‘CC’). However, underlying mechanisms are unknown. We aimed to examine potential mechanisms explaining the association between rs6897932 and CD4+ T cell recovery. Ten ‘TT’ and 10 ‘CC’ HIV-infected individuals matched on gender, age, and nadir and current CD4+ T cell counts were included in a cross-sectional study. ‘TT’ individuals had higher proportion of CD4+ T cells expressing pSTAT5 compared to ‘CC’ individuals after stimulating with IL-7, especially when co-stimulated with soluble IL7-RA (sIL-7RA). Furthermore, ‘TT’ individuals had a higher proportion of proliferating CD4+ T cells after 7 days of culture with IL-7 + sIL-7RA compared to ‘CC’ individuals. No differences between ‘TT’ and ‘CC’ in binding of biotinylated IL-7 were found. In conclusion, increased signal transduction and proliferation in response to IL-7 was found in ‘TT’ compared to ‘CC’ HIV-infected individuals providing a mechanistic explanation of the effect of rs6897932 T-allele on CD4+ T cell recovery in HIV infection.

Untreated HIV infection is characterized by a progressive loss of CD4+ T cells leading to AIDS and death^1^. Initiation of combination antiretroviral treatment (cART) usually results in suppression of viral replication followed by immune recovery with increasing CD4+ T cell count^1^,^2^,^3^. However, great variation in the rate of CD4+ TCD4+ T cell recovery is observed, and approximately 20% of individuals initiating cART do not achieve optimal immune reconstitution with CD4+ T cell count above 500 cells/μL two years after initiation of cART with increased risk of morbidity and mortality^3^.

Interleukin-7 (IL-7) and the IL-7 receptor (IL-7R) are essential for the CD4+ T cell homeostasis by promoting survival, proliferation, and de novo production of T cells^4^. We and others have previously described that a single nucleotide polymorphism (SNP, rs6897932, T/C) in the gene encoding CD127 (IL-7RA) was associated with faster CD4+ T cell recovery after initiating cART in HIV-infected individuals^5^,^6^,^7^. Thus, in a cohort of 1,683 HIV-infected individuals, T-allele homozygosity in rs6897932 compared to carrying a C-allele resulted in improved CD4+ T cell recovery (130%) after 6 and 12 months of suppressive cART^5^. However, the mechanisms by which rs6897932 T-allele homozygosity enhances CD4+ T cell recovery after initiation of cART remain unclear, and since IL-7 has been suggested as adjuvant treatment in HIV infection unravelling the mechanisms of its effects are of great importance.

Possible mechanisms of rs6897932 include altered affinity of the IL-7R, altered expression of IL-7RA, altered level of soluble IL-7RA (sIL-7RA), altered intracellular signaling, or altered proliferation and viability of CD4+ T cells. rs6897932 is located in the transmembrane region of IL-7RA, and the rs6897932 T-allele is associated with a decreased plasma level of soluble IL-7RA (sIL-7RA) compared to the rs6897932 C-allele^8^,^9^,^10^. This has been suggested to explain the effect of rs6897932 on CD4+ T cell recovery. However, *in vivo* evidence do not support an effect of sIL-7RA on CD4+ T cell count^11^, and *in vitro* studies found increased viability and proliferation of murine and human cells cultured with IL-7 plus sIL-7RA compared to IL-7 alone^9^,^12^. This questions whether altered concentration of sIL-7RA explains the effect of rs6897932 on CD4+ T cell recovery. To our knowledge, no studies have investigated whether rs6897932 affects the affinity of IL-7R, the intracellular signaling of IL-7R, or the IL-7R response in T cells. We hypothesized that T-allele homozygosity (‘TT’) compared to C-allele homozygosity (‘CC’) in rs6897932 increases the binding of IL-7, increases the intracellular signaling of the IL-7R, and increases the IL-7R response in CD4+ T cells. To investigate this, the binding of biotinylated IL-7 to CD4 + CD127+ T cells and the intracellular expression of phosphorylated Signal transducer and activator of transcription 5 (pSTAT5) in CD4+ T cells as well as CD4+ T cell proliferation after IL-7 stimulation were examined in ‘TT’ HIV-infected individuals compared to ‘CC’ HIV-infected individuals in an *in vitro* setting. Furthermore, the effect of sIL-7RA in combination with IL-7 was examined by repeating the investigations with increasing levels of sIL-7RA.

## Results

### Study participants

In the Danish HIV-infected population ‘TT’ is present in 7.4% and ‘CC’ in 54.6%^5^. A total of 10 ‘TT’ HIV-infected individuals were randomly selected from the Danish HIV Cohort Study and 10 ‘CC’ individuals were selected to match on gender, age, CD4 nadir, current CD4+ T cell count, and time on cART (Table 1). All participants were men, the median age was 52.8 years (48.3–61.3), and no clinical differences comparing the two groups were present (Table 1). Furthermore, no differences in plasma concentrations of IL-7 and sIL-7RA between the two groups were found (Table 1).

### Binding of IL-7 to IL-7R

**Figure 1:**
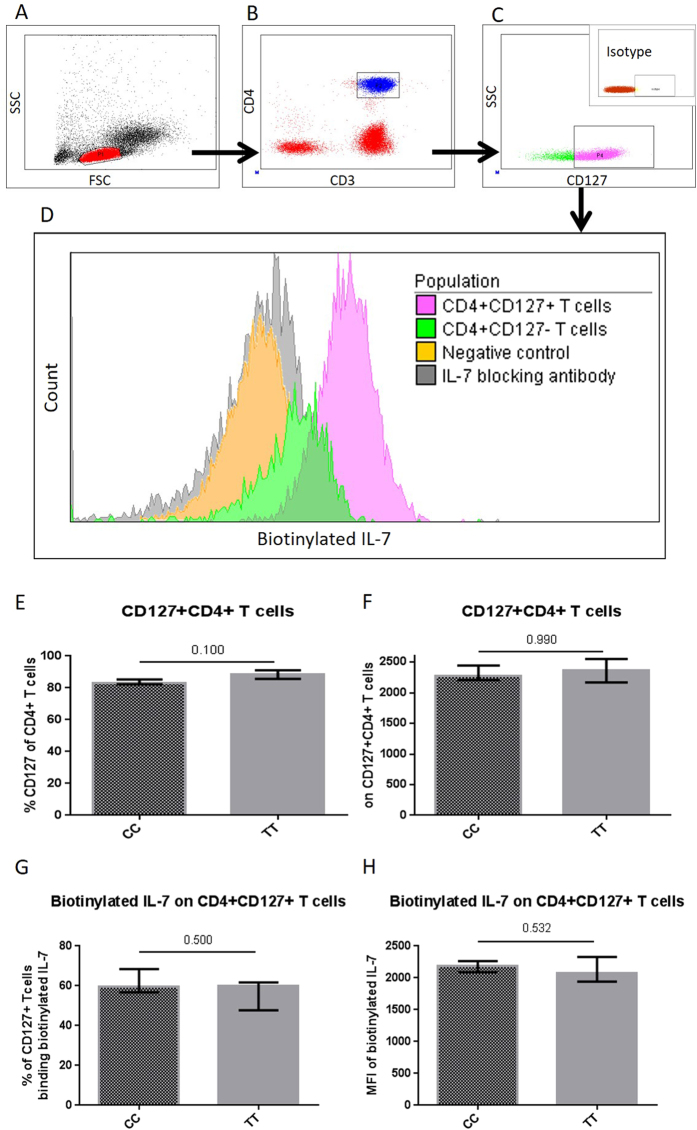
Binding of biotinylated IL-7. Representative plots illustrating gating strategy of (**A**) PBMC, (**B**) CD4+ T cells, and (**C**) CD4 + CD127+ T cells including isotype control plot for the CD127 gate. The binding of biotinylated IL-7 is illustrated in (**D**). Increased binding of biotinylated IL-7 was seen on CD4 + CD127+ T cells compared to CD4 + CD127- T cells. Furthermore, the assay was tested by including a negative control, and IL-7 blocking antibody (**D**). The proportion of CD4+ T cells expressing CD127 and the MFI of CD127 was compared in ‘TT’ versus ‘CC’ (**E**,**F**). The bound biotinylated IL-7 on CD4 + CD127+ T cells was compared in ‘TT’ versus ‘CC’ in regards of (**G)** proportion of CD4 + CD127+ T cells binding biotinylated IL-7 and (**H**) MFI of bound biotinylated IL-7 on CD4 + CD127+ T cells.

Binding of biotinylated IL-7 (btIl-7) to CD127+ T cells was tested by measuring the proportion of CD127 + CD4+ T cells positive for btIL-7 and the MFI of btIL-7 on these cells. A non-reactive biotinylated protein was used as negative control (Fig. 1D), and adding blocking anti-IL-7 antibody demonstrated the specificity of the assay (Fig. 1D). Furthermore, reduced binding of btIL-7 was observed on CD4 + CD127- T cells compared to the CD4 + CD127 + T cells (Fig. 1D). No differences between the two groups were found when comparing the proportion of CD127 + CD4+ T cells and the MFI of CD127 on CD127 + CD4+ T cells (Fig. 1E,F). The proportion of CD127 + CD4+ binding btIL-7 was comparable in the two groups (‘CC’ (59.8% (56.8–68.5) vs. ‘TT’ (60.1% (47.8–61.8), p = 0.500), Fig. 1G). Similarly, no difference in the MFI of btIL-7-positive CD127 + CD4+ T cells (p = 0.532) was found (Fig. 1H).

### Intracellular signaling

**Figure 2:**
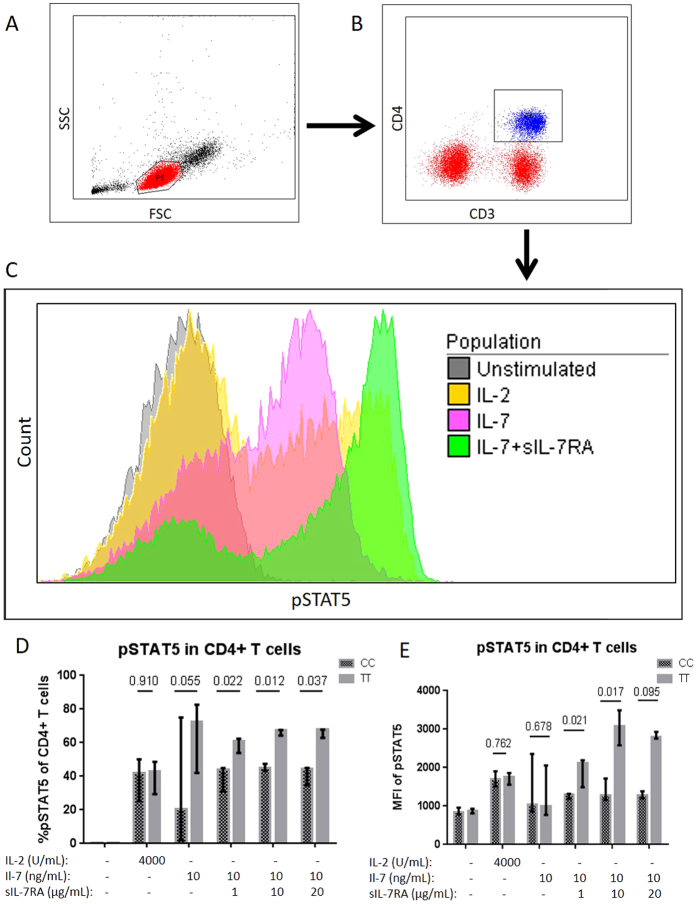
Intracellular signaling by pSTAT5. Representative plots illustrating gating strategy of (**A**) PBMC, (**B**) CD4+ T cells, and (**C**) pSTAT5 expression in unstimulated and stimulated with IL-2, IL-7, IL-7 + sIL-7RA (1 to 1000). The pSTAT5 expression was compared in ‘TT’ versus ‘CC’ in regards of (**D**) proportion of pSTAT5 + CD4+ T cells and (**E**) MFI of pSTAT5 on CD4+ T cells.

To investigate the intracellular signal transduction of IL-7R after stimulation with IL-7 and IL-7 + sIL-7R, the proportion of T cells expressing pSTAT5 was assessed. As control, the proportion of T cells expressing pSTAT5 after stimulation with IL-2 was assessed. ‘TT’ individuals had a borderline significantly higher proportion of pSTAT5 + CD4+ T cells (72.3% (82.7–42.0)) vs. ‘CC’ individuals (20.5% (2.0–75.0), p = 0.055) after stimulating with IL-7 alone, while no difference in pSTAT5 MFI was found (Fig. 2D,E). However, stimulating with both IL-7 and sIL-7RA disregarding the concentration of sIL-7RA resulted in both higher proportion of pSTAT5 + CD4+ T cells in ‘TT’ individuals (IL-7 + sIL-7RA (1 to 100) p = 0.022, IL-7 + sIL-7RA (1 to 1000) p = 0.012, and IL-7 + sIL-7RA (1 to 2000) p = 0.037, Fig. 2D) and higher MFI of pSTAT5 positive CD4+ T cells in ‘TT’ individuals (Fig. 2E). No differences between the two groups in the proportion of pSTAT5 + CD4+ T cells or MFI of pSTAT5 positive cells after IL-2 stimulation were found (Fig. 2D,E).

The result of higher proportion of pSTAT5 + CD4+ T cells when stimulating with both IL-7 and sIL-7RA compared to IL-7 alone was validated in four additional independent HIV-samples (Supplementary Material, Fig. 1). Furthermore, no effect of stimulating with sIL-7RA alone on the proportion of pSTAT5 + CD4+ T cells was found (Supplementary Material, Fig. 1).

### Proliferation assay

**Figure 3:**
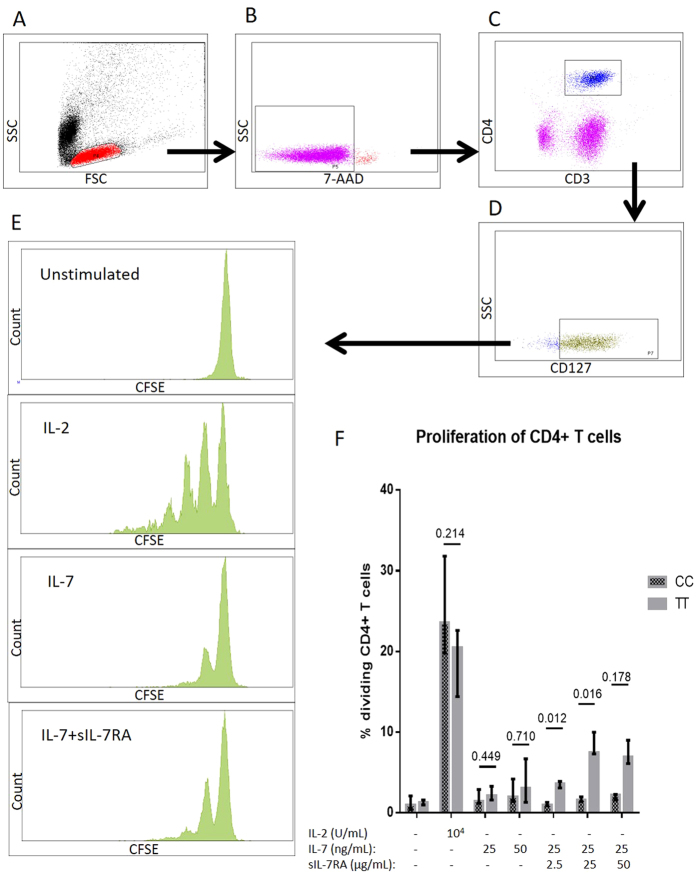
Proliferation assay. Representative plots illustrating gating strategy of (**A**) PBMC, (**B**) viable cells, (**C**) CD4+ T cells, (**D**) CD4 + CD127+ T cells, and (**E**) the proliferation of unstimulated cells, and cells stimulated with IL-2, IL-7, and IL-7 + sIL-7RA (1 to 2000). (**F**) The proportions of CD4+ T cells that have made at least one cell division were compared in ‘TT’ versus ‘CC’.

The viability and proliferation of CD4+ T cells after stimulation with IL-7, IL-7 + sIL-7RA, and IL-2 were compared between the two groups (Fig. 3A–F). No difference in proportion of proliferating cells between the two groups was observed after culture with IL-7 alone, but ‘TT’ individuals presented with higher proportions of proliferating CD4+ TCD4+ T cells after culture with IL-7 in combination with sIL-7RA (IL-7 + sIL-7RA (1 to 100), p = 0.012, and IL-7 + sIL-7RA (1 to 1000), p = 0.016), while no statistical difference between the two groups was observed combining IL-7 with sIL-7RA in a ratio of 1 to 2000 (p = 0.178, Fig. 3F). In contrast, no differences in proportion of proliferating cells between the two groups were observed after culture with IL-2 (Fig. 3F). Additionally, we investigated the proportion of proliferating CD4 + CD127+ T cells after stimulating with IL-7, IL-7 + sIL-7RA, and IL-2 demonstrating similar results (data not shown). No differences between ‘TT’ and ‘CC’ individuals were found in the viability of PBMC regardless of the stimuli (Supplementary Material, Fig. 2).

Finally, we investigated the proportion of proliferating CD127+ T cells after culture with PHA. ‘CC’ individuals presented with higher proportion of proliferating CD4 + CD127+ T cells (76.4% (46.7–79.8)) compared to ‘TT’ individuals (32.1% (23.3–45.4), p = 0.007) after PHA stimulation.

## Discussion

Improved CD4+ T cell recovery after cART initiation in HIV-infected individuals being T-allele homozygote in rs6897932 compared to individuals carrying a C-allele has been reported^5^,^6^,^7^. The underlying mechanisms, however, remain unclear. In this cross-sectional study, we demonstrated that ‘TT’ compared to ‘CC’ HIV-infected individuals had increased intracellular signaling after stimulation with IL-7 measured as the proportion of phosphorylation of STAT5 in CD4+ T cells, especially after stimulation with both IL-7 and sIL-7RA. Furthermore, ‘TT’ individuals had increased proliferation of CD4+ T cells after culture with IL-7 plus sIL-7RA. No differences between ‘TT’ and ‘CC’ individuals were found in IL-7 binding to IL-7R and viability of PBMC. These data suggest enhanced intracellular signaling and increased proliferation to explain faster CD4+ T cell recovery after initiation of cART in ‘TT’ HIV-infected individuals^5^.

rs6897932 is a nonsynonymous SNP substituting threonine with isoleucine in exon 6 located in the transmembrane region of IL-7RA^8^. The C-allele has been described to cause alternative splicing of IL-7RA by augmenting an exonic splicing silencer (ESS) resulting in increased ratio between sIL-7RA and membrane-bound IL-7RA (mbIL-7RA)^8^. Increased plasma sIL-7RA has previously been suggested to explain the effect of rs6897932^6^,^8^,^9^,^10^. However, in the present study ‘TT’ and ‘CC’ individuals did not differ in plasma sIL-7RA. In contrast, the results indicate that the effect of rs6897932 is driven by an increased response of IL-7R in ‘TT’ to IL-7 and sIL-7RA. Importantly, increased IL-7R response in CD4+ T cell in ‘TT’ is in line with the reported faster CD4+ T cell recovery in ‘TT’ HIV-infected individuals^5^. This is further supported by an association between increased IL-7 responsiveness in T cells and higher CD4+ T cell count in HIV-infected individuals^13^. The molecular explanation of an altered response of IL-7RA in ‘TT’ compared to ‘CC’ cannot be provided by the current study, and whether rs6897932 directly modulates the response of IL-7RA or indirectly by linkage disequilibrium with another allele remains uncertain. A higher affinity of IL-7R in ‘TT’ compared to ‘CC’ may explain the difference in IL-7R response including the accentuated difference when stimulating with both sIL-7RA and IL-7. In the present study, however, MFI of biotinylated IL-7 on CD127+ T cells was determined, and no evidence to support altered binding between IL-7 and CD127 was found. It cannot be excluded that use of ligand binding assays may have provided additional information.

The effect of rs6897932 on the T cell response to IL-7 and sIL-7RA was strengthened by the fact that no difference in pSTAT5 expression or proliferation were found between ‘TT’ and ‘CC’ individuals after stimulating with IL-2. Interestingly, a higher PHA-induced proliferative response in ‘CC’ individuals compared to ‘TT’ individuals was found. While IL-7 and IL-2 are naturally occurring *in vivo*, PHA is an unnatural stimulus. Thus, the higher PHA-induced response in ‘CC’ individuals may indicate that different activation pathways are more potent in ‘CC’ compared to ‘TT’ individuals. The increased pSTAT5 expression observed when stimulating with both IL-7 + sIL-7RA compared to IL-7 alone is in contrast with two previous studies^9^,^11^. Thus, we retested our setup in an independent group of four HIV-infected individuals with similar results, and further demonstrated that sIL-7RA alone had no effect on pSTAT5 expression highlighting the importance of the combination of IL-7 and sIL-7RA. The result may seem puzzling, but could indicate enhancement of the IL-7 response when stimulating with IL-7 + sIL-7RA. Future studies to elucidate the mechanism are warranted.

In previous studies using bot murine and human T cells, co-stimulation with sIL-7RA increased IL-7 induced proliferation^9^,^12^, which is in accordance with findings in the present study. However, contrasting findings have also been shown^9^,^11^. The intuitively paradoxical effect of sIL-7RA on T cell proliferation may be caused by diminished IL-7 consumption of the cells when stimulating with both IL-7 and sIL-7RA thereby prolonging the period with sufficiently effective IL-7 level^9^,^12^. This requires higher affinity of the membrane-bound receptor compared to the soluble receptor and has also been found for IL-15 and sIL-15^14^. Thus, the source of sIL-7RA in studies may play a role due to differences in affinity such as in native sIL-7RA compared to sIL-7RA combined with a human Fc (sIL-7RA-Fc)^9^ which was used in the current study. Furthermore, it has to our knowledge not been investigated, whether the isoform of sIL-7RA in plasma differs between ‘TT’ and ‘CC’.

Previously, we have shown an association between the rs6897932 T-allele and increased mortality during untreated HIV infection^15^. Thus, in a cohort of 152 untreated HIV-infected individuals carrying a T-allele compared to ‘CC’ in rs6897932 resulted in a 2.56 hazard ratio of mortality suggesting another effect of IL-7 in HIV infection. Recently, supplementary recombinant human (rh) IL-7 failed to reduce the HIV reservoir by HIV reactivation^16^, as rhIL-7 administration increased the HIV reservoir by inducing proliferation of central memory CD4+ T cells containing HIV-DNA^16^. Thus, an active and potent IL-7R response in ‘TT’ individuals may be advantageous to increase CD4+ T cell recovery during suppressive cART, but a disadvantage during untreated HIV infection, as the effect on CD4+ T cells accelerate the viraemia and thereby possibly also disease progression. Supplementary rhIL-7 administration in HIV-infected individuals may not be used as HIV-reactivation agent to eradicate HIV^16^, but rhIL-7 increases CD4+ T cell count and seem well-tolerated^17^,^18^. Thus, administration of rhIL-7 as a supplement to suppressive cART in immunological non-responders seem promising^19^. In future clinical studies, it would be interesting to consider the potentiating effect of sIL-7RA on IL-7 as well as to investigate whether rs6897932 affects the rhIL-7 response *in vivo*.

The present study is limited by a small study cohort, and the results were not adjusted for multiple comparisons. Furthermore, ‘CT’ individuals were not included, and it would have been valuable to examine whether the reported differences between ‘CC’ and ‘TT’ individuals can be explained by a dominant or recessive effect of the T-allele. To our knowledge, comparable studies have not been undertaken, and the results need to be confirmed in independent studies. Especially, studies in humanized mice or a cell line expressing ‘CC’, ‘CT’, and ‘TT’ would provide new insight into the physiological relevance of rs6897932 as well as into the molecular mechanisms underlying the described association between rs6897932 and IL-7R response on CD4+ T cells. Importantly, the association between rs6897932 and IL-7R response may not be explained by rs689732 in itself, but by linkage disequilibrium with other SNPs in IL-7RA or other genes such as genes encoding other cytokine receptors or intracellular signaling pathways proteins^20^. Finally, it would be interesting to investigate the effect of rs6897932 on IL-7R function in individuals with multiple sclerosis or during post-transplant immune recovery where rs6897932 already has been associated with clinical endpoints^21^,^22^,^23^,^24^.

In conclusion, ‘TT’ individuals have an increased IL-7RA response in CD4+ T cells compared to ‘CC’ individuals which was shown by increased intracellular signal transduction and increased proliferation of CD4+ T cells when stimulating with IL-7 and sIL-7RA. This provides new insight to the underlying mechanisms of the effect of rs6897932 on CD4+ T cell recovery as well as the role of sIL-7RA in HIV-infected individuals.

## Methods

### Study population

A total of 20 HIV-infected individuals were included in this cross sectional study. Inclusion criteria were suppressive cART for at least 12 months (<20 copies/mL), Caucasian ethnicity, and age between 18 and 70 years. Individuals available for inclusion were identified by having a valid test of rs6897932 in the Danish HIV Cohort Study demonstrating ‘CC’ or ‘TT’^5^. Participants were included in two groups according to the allele distribution in rs6897932 (‘CC’ or ‘TT’). All participants were enrolled from Department of Infectious Diseases, Rigshospitalet, Copenhagen University Hospital. Informed consent was obtained in writing and verbally from all participants, and the study was performed in accordance with the ethical guidelines of the 1975 Declaration of Helsinki and approved by the Local Ethical Committee (H-1-2014-049) and the Danish Data Protection Agency.

### Blood samples

Blood collected in heparin tubes (Becton Dickinson (BD), Franklin Lakes, NJ, USA) was used for isolation of peripheral blood mononuclear cells (PBMC) by density gradient centrifugation, 400 g for 25 minutes (Histopaque; Sigma-Aldrich, St. Louis, MO, USA). Blood collected in ethylenediamine tetraacetic acid (EDTA) tubes (BD) was used for plasma isolation. Plasma was isolated within 30 min after collection of blood samples and immediately stored at −80 °C.

### Binding of IL-7 to IL-7R on T cells

Fluorokine biotinylated human IL-7 (NF700, R&D Systems, Abingdon, UK) was used to determine the binding of IL-7 to CD4 + CD127+ T cells according to manufacturer’s instruction. In brief, biotinylated IL-7 and 10^5^ PBMC suspended in 25 μL PBS were incubated at 5 °C for 90 min followed by incubation with avidin-FITC (R&D Systems) and the monoclonal antibodies CD3-APC-H7, CD4-PeCy7, and CD127-AF647 (all purchased from BD), and assessed by flow cytometry. A biotinylated non-reactive protein (soybean trypsin inhibitor, R&D systems) was used as a negative control, and specificity was tested by adding a polyclonal IL-7 blocking antibody (R&D) (representative plots in Fig. 1A–D).

### Intracellular signaling

Intracellular signaling was assessed by determining the phosphorylation of STAT5 by flow cytometry. In brief, 500 μL of PBMC suspension (10^6^ cells/μL) was incubated for 15 minutes at 37 °C with 4000 U/mL IL-2 (kindly provided by N. Ødum, Copenhagen University, Copenhagen) or 10 ng/mL IL-7 (Peprotech, Stockholm, Sweden). Furthermore, PBMC from a total of ten participants (five ‘CC’ and five ‘TT’) were stimulated with 10 ng/mL IL-7 in combination with sIL-7RA (IL-7 + sIL-7RA) in three different ratios: 1 to 100, 1 to 1000, or 1 to 2000 (sIL-7RA was purchased from R&D Systems, Abingdon, UK). The stimulated PBMC were fixated with fixation buffer (BD), incubated with monoclonal antibodies (CD3-FITC and CD4-APC-H7) (BD), permeabilized with permeabilization buffer (BD), incubated for 30 min with antibody pSTAT5-AF647 (BD), and immediately assessed by flow cytometry (representative plots in Fig. 2A–C).

### Proliferation assay

PBMC were incubated with CFSE (1.5 μM) for 10 minutes at 37 °C. PBMC (2 × 10^6^ cells/μL) were cultured for seven days with autologous serum and with either 25 or 50 ng/mL IL-7 (Peprotech), 10^4^ U/mL IL-2 or 5 μg/mL phytohaemagglutinin (PHA) (Sigma-Aldrich, St. Louis, MO, USA). Furthermore, in a total of ten participants (five ‘CC’ and five ‘TT’) proliferation was assessed in cells cultured with IL-7 (25 ng/ml) plus sIL-7RA in three different ratios (1 to 100, 1 to 1000 or 1 to 2000) in PBMC). After seven days the cells were incubated with antibodies (CD3-APC-H7, CD4-PeCy7, CD127-AF647, and 7-AAD) (all purchased from BD) and assessed by flow cytometry. The data are given as the proportion of CD4+ T cells and the proportion of CD4 + CD127+ T cells that has made at least one cell division (representative plots in Fig. 3A–E).

### ELISA

Plasma concentrations of IL-7 and sIL-7RA were determined by competitive ELISA kits (NeoBiolab, Woburn, MA, USA) according to manufacturer’s instructions and analyzed on ELISA reader (BMG Labtech Inc., Cary, NC, USA). All standards and samples were measured in duplicates.

### Statistics

The Mann-Whitney U test was used to evaluate differences between the two groups of HIV-infected individuals. All data are presented as medians and interquartile range (IQR). All statistics were performed using SAS (Version 9.2, SAS Institute, Copenhagen, Denmark), and graphs were produced in GraphPad Prism 6.0 (GraphPad Software, La Jolle, CA, USA).

## Additional Information

**How to cite this article:** Hartling, H. J. *et al*. Gene variation in IL-7 receptor (IL-7R)α affects IL-7R response in CD4+ T cells in HIV-infected individuals. *Sci. Rep.* **7**, 42036; doi: 10.1038/srep42036 (2017).

**Publisher's note:** Springer Nature remains neutral with regard to jurisdictional claims in published maps and institutional affiliations.

## Supplementary Material

Supplementary Material

## Figures and Tables

**Table 1:** Clinical characteristics.

rs6897932	TT, N = 10	CC, N = 10	P-value
Men/Women, N	10/0	10/0	NA
Age, years	51.7 (44.9–57.9)	53.1 (49.7–62.3)	0.427
Current CD4+ T cell count, cells/μL	660 (620–720)	610 (550–790)	0.820
Nadir CD4+ T cell, cells/μL	185 (74–260)	190 (44–290)	0.999
Time on cART, years	15.9 (8.2–19.2)	16.8 (16.0–18.8)	0.970
Plasma IL-7, pg/mL	50.5 (44.7–65.9)	55.4 (48.8–57.6)	0.791
Plasma sIL-7RA, pg/mL	1260 (863–1434)	1034 (856–1207)	0.545

Comparisons of clinical characteristics and plasma concentration of IL-7 and sIL-7RA in 10 HIV-infected individuals with T-allele homozygosity in rs6897932 (TT) and 10 HIV-infected individuals with C-allele homozygosity in rs6897932 (CC).

All data are presented as median (interquartile range; IQR). All participants had fully suppressed viral replication (<20 copies/mL). NA: not applicable
