# Supplementary material for: Gene variation in IL-7 receptor (IL-7R)α affects IL-7R response in CD4+ T cells in HIV-infected individuals
Source: Sci Rep. 2017 Feb 9;7:42036. doi: 10.1038/srep42036 (PMC5299473; doi:10.1038/srep42036)
Supplement: Supplementary Material [file srep42036-s1.pdf]

Gene variation in IL-7 receptor (IL-7R)α affects IL-7R response in CD4+ T cells in HIV-infected individuals

Hans Jakob Hartling [*,†]

Lars P Ryder [†]

Henrik Ullum [†]

Niels Ødum [‡]

Susanne Dam Nielsen [*]

*) *Viro-Immunology Research Unit, Department of Infectious Diseases, Rigshospitalet, Copenhagen University Hospital, Denmark*

†) *Department of Clinical Immunology, Rigshospitalet, Copenhagen University Hospital, Denmark*

‡) *Department of Immunology and Microbiology, Faculty of Health and Medical Sciences, University of Copenhagen, Copenhagen, Denmark,*

Corresponding author: Susanne Dam Nielsen; telephone: +45 35450859; sdn@dadlnet.dk

Supplementary materials

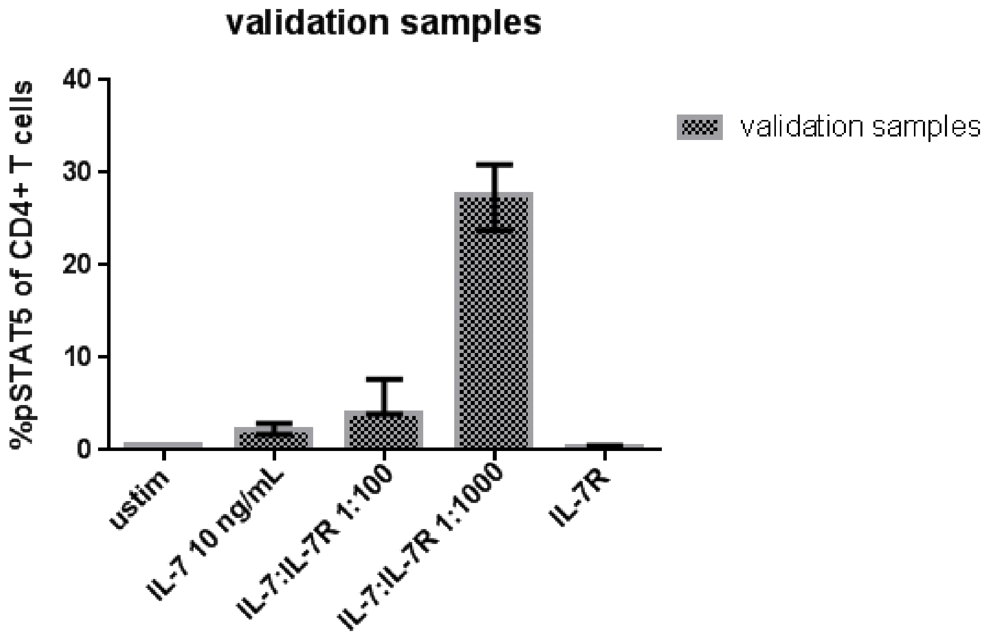

Figure 1, supplementary material. We validated our results in four additional samples from not previously examined HIV-infected individuals demonstrating similar result of higher proportion of pSTAT5+CD4+ T cells when stimulating with both IL-7 and sIL-7RA. Furthermore, no effect of stimulating with sIL-7RA alone was found.

Supplementary materials

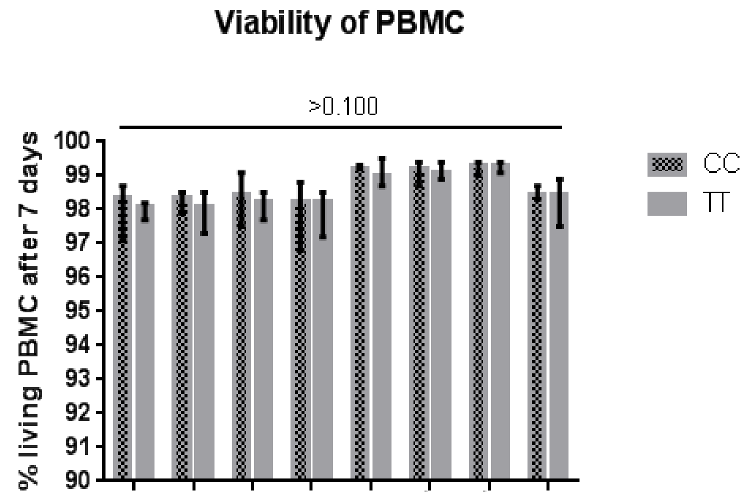

IL-2 (U/mL):	-	10^4	-	-	-	-	-	-
IL-7 (ng/mL):	-	-	25	50	25	25	25	-
sIL-7RA (µg/mL):	-	-	-	-	2.5	25	50	-
PHA (µg/mL):	-	-	-	-	-	-	-	5

Figure 2, supplementary material. Viability of PBMC
The viability after 7 days of culture was compared in 'TT' versus 'CC'. Percent of PBMC negative for 7-AAD was used as a measurement of living cells. Please notice the y-axis starts at 90 %.
